# The role of primary care during the pandemic: shared experiences from providers in five European countries

**DOI:** 10.1186/s12913-023-09998-0

**Published:** 2023-10-02

**Authors:** Markus Kraus, Christoph Stegner, Miriam Reiss, Monika Riedel, Anne Sofie Børsch, Karsten Vrangbaek, Morgane Michel, Kathleen Turmaine, Borbála Cseh, Csaba László Dózsa, Roberto Dandi, Angelo Rossi Mori, Thomas Czypionka

**Affiliations:** 1 Institute for Advanced Studies (IHS), Josefstädter Straße 39, Vienna, 1080 Austria; 2 University of Copenhagen, Øster Farimagsgade 5, Copenhagen K, 1353 Denmark; 3 Université Paris Cité, ECEVE, UMR 1123, 10 avenue de Verdun, Inserm, Paris, 75010 France; 4 Unité d’épidémiologie clinique, Assistance Publique-Hôpitaux de Paris, Hôpital Robert Debré, 48 boulevard Sérurier, Paris, 75019 France; 5 University of Miskolc, Egyetem út 1, Miskolc-Egyetemváros, 3515 Hungary; 6 Luiss Business School, Via Nomentana 216, Roma, 00162 RM Italy; 7 Institute for Research on Population and Social Policies, Via Palestro 32, Roma, 00185 Italy; 8 London School of Economics and Political Science, Houghton Street, London, WC2A 2AE UK

**Keywords:** COVID-19, Primary care, Service delivery, Resilience, Pandemic preparedness

## Abstract

**Background:**

The COVID-19 pandemic necessitated wide-ranging adaptations to the organisation of health systems, and primary care is no exception. This article aims to collate insights on the role of primary care during the pandemic. The gained knowledge helps to increase pandemic preparedness and resilience.

**Methods:**

The role of primary care during the pandemic in five European countries (Austria, Denmark, France, Hungary, Italy) was investigated using a qualitative approach, namely case study, based on document analysis and semi-structured interviews. In total, 31 interviews were conducted with primary care providers between June and August 2022. The five country case studies were subjected to an overarching analysis focusing on successful strategies as well as gaps and failures regarding pandemic management in primary care.

**Results:**

Primary care providers identified disruptions to service delivery as a major challenge emerging from the pandemic which led to a widespread adoption of telehealth. Despite the rapid increase in telehealth usage and efforts of primary care providers to organise face-to-face care delivery in a safe way, some patient groups were particularly affected by disruptions in service delivery. Moreover, primary care providers perceived a substantial propagation of misinformation about COVID-19 and vaccines among the population, which also threatened patient-physician relationships. At the same time, primary care providers faced an increased workload, had to work with insufficient personal protective equipment and were provided incongruous guidelines from public authorities. There was a consensus among primary care providers that they were mostly sidelined by public health policy in the context of pandemic management. Primary care providers tackled these problems through a diverse set of measures including home visits, implementing infection control measures, refurbishing used masks, holding internal meetings and relying on their own experiences as well as information shared by colleagues.

**Conclusion:**

Primary care providers were neither well prepared nor the focus of initial policy making. However, they implemented creative solutions to the problems they faced and applying the learnings from the pandemic could help in increasing the resilience of primary care. Attributes of an integrated health system with a strong primary care component proved beneficial in addressing immediate effects of the pandemic.

**Supplementary Information:**

The online version contains supplementary material available at 10.1186/s12913-023-09998-0.

## Introduction

The COVID-19 pandemic along with the strategies taken to limit its spread continue to have a profound impact on health systems globally. Primary care is a cornerstone in achieving the goals envisioned in the 2018 Astana Declaration [1] and also the first line of defence in a pandemic [2, 3]. Primary care is essential in addressing the health burden stemming from the pandemic [4].

Previous research on experiences and learnings have been devoted to specific population groups or early phases of the pandemic, most of it focusing on single countries [5–28]. For example, the implementation and usage of telehealth as one pandemic response was analysed in many countries, including Canada [7, 20], Sweden [10], the UK [23, 25] and the USA [16, 19]. Generally, these studies found an increase in telehealth utilisation and improvements in access to care for some patients but deteriorations for other, more vulnerable groups. Advantages like decreased infection transmission risk and reduced travel time must be weighed against disadvantages such as lack of physical examination and difficulties in establishing trust with new patients. Several authors also documented an adverse impact on burnout and an increased workload of primary care providers in, e.g., Belgium [26], Germany [5, 9, 15], Spain [11, 22], the UK [6] and the USA [18].

Similarly, there is also a considerable amount of research investigating the effects of the pandemic on specific population groups like children [29–35], the elderly [36–42], patients with chronic diseases [43–47], migrants and refugees [48–51] and people who use drugs [52–54]. For example, Bode et al. [29], Lee et al. [32] and Zhong et al. [35] observed a decrease in (routine) childhood vaccinations post-pandemic compared to pre-pandemic. Although primary care providers continued to offer vaccinations, parents were seemingly reluctant to visit primary care practices to avoid potential infections. Moreover, Franzosa et al. [38] and Gorbenko et al. [39] analysed the effects of the pandemic on elderly persons in home-based primary care. They found that providers were required to implement extensive and rapid adaptations to a traditionally hands-on model of care. In particular, they introduced both patient-centred (e.g., screenings for loneliness, anxiety and depression, discussing challenges) and practice-centred measures (e.g., emotional support for staff, increased team meetings) to minimise disruptions to care delivery.

Building on an online survey in 38 countries, a series of articles investigates the reorganisation of primary care during the pandemic [55]. For example, Groenewegen et al. [56] investigated task changes of primary care staff in course of the pandemic and found an increase in responsibilities of staff as well as a greater involvement in outreach to vulnerable patients. Windak et al. [57] focused on the appropriateness of primary care infrastructure and observed limitations to provide high-quality and safe care in about 6 out of 10 practices. Petrazzuoli et al. [58] explored differences between rural and urban practices and noted distinctions, among others, in patient characteristics, likeliness of using video consultations and prescribing practices.

Little to no attention has been paid to in-depth cross-country analyses covering multiple aspects using qualitative methods. An exception is a series of articles by Wanat et al. [3, 59, 60]. Covering eight European countries, these articles investigated primary care providers’ perceptions on aspects of service delivery [60], personal risk and testing [59] and changing patient-physician relationships [3]. However, as the interviews were conducted in the early periods of the pandemic (between April and July 2020), they are limited to the learnings accumulated up to that point. Although learnings from the early phase of the pandemic are certainly still relevant and useful, a thorough understanding of the impacts of the pandemic on primary care requires an up-to-date appraisal of the existing knowledge. In order to contribute towards filling this gap in the literature, this article aims to collate insights from the various impacts of the pandemic on primary care in five European countries (Austria, Denmark, France, Hungary and Italy) up to two and a half years after the declaration of the pandemic. The analysis was led by the underlying research question: “What was the role of primary care systems during the COVID-19 pandemic and what can be done to increase pandemic preparedness and resilience?”. By analysing five European countries and encompassing a time span until summer 2022, this article strives to provide the first comprehensive analysis focusing on successful strategies, as well as gaps and failures regarding pandemic management in primary care. Thereby, it offers a broader set of European experiences for mutual learning.

This research is part of the PERISCOPE project (Pan-European Response to the ImpactS of COVID-19 and future Pandemics and Epidemics; https://periscopeproject.eu), a research project funded by the Horizon 2020 programme of the European commission. Among others, the project aims to analyse preparedness and adaptive capacity of health systems with regards to COVID-19, assess the impact of the outbreak and policy measures on health systems and draw lessons from these experiences to improve health system resilience. Different definitions of health system resilience have been put forward, which the World Health Organization (WHO) [61] summarises as ‘the ability of all actors and functions related to health to collectively mitigate, prepare, respond and recover from disruptive events with public health implications, while maintaining the provision of essential functions and services and using experiences to adapt and transform the system for improvement’. For an in-depth discussion on resilience see, for example, OECD [62] or WHO [61]. For the purpose of our analysis, resilience is referring to the COVID-19 pandemic as the disruptive event, serious threat or hazard.

## Method

### Country case studies

The methodological approach of a case study [63–67] was chosen to investigate pandemic preparedness of primary care systems in selected European countries. A case study scientifically investigates a real-life phenomenon in-depth and within its environmental context. Such a case can be an individual, a group, an organization, an event, a problem or an anomaly [68]. For case analyses, the emphasis in data collection is on interviews, archives and (participant) observation resulting in a detailed case description [69, 70]. In the current article the primary care system of a country represents a case and the data are collected by means of document analysis and semi-structured interviews.

The country case studies aimed to identify successful strategies and lessons learnt as well as gaps and failures regarding pandemic management in primary care. In the country case studies, a special focus was put on aspects of service delivery (e.g., in which ways quality of and access to care were affected), on information provided to primary care providers and patients, as well as on guidelines/regulations for primary care providers (e.g., how primary care providers perceived the various COVID-19-specific guidelines and regulations that were relevant for primary care, both from authorities and from professional associations). These aspects were prioritised based on gaps identified in the literature in an (ongoing) scoping review registered on the Open Science Framework (identifier: osf-registrations-93tf6-v1).

Five countries were individually investigated by means of a country case study by researchers from the respective countries following a common template. The selection of countries for the case studies was driven by five main principles: (1) different degrees to which countries were affected by different COVID-19 waves, (2) geographic coverage of member states of the European Union, i.e., one country from Western, Central, Northern, Eastern and Southern Europe, respectively, reflecting different cultural aspects and social norms, (3) balanced mix of countries in terms of population characteristics, (4) balanced mix of countries with different primary care models (single practice vs. group practice/primary health care centres, gate keeping, primary care vs. secondary care), (5) balanced mix of countries with different health systems, more precisely, Beveridge vs. Bismarck model of health care financing. The application of these criteria resulted in the selection of the following five countries: Austria, Denmark, France, Hungary and Italy. Table 1 provides an overview of population and health system characteristics of the selected countries.

**Table 1:** Country characteristics

	Population characteristics					Health system characteristics							
Country	Population size (2020)^1^	Population density (2020)^1^	Population aged 65 or older (2020)^1^	Healthy life years at birth (2020)^1^	Life expectancy at birth (2020)^1^	General type of health care system	Predominant primary care model	Primary care system has gate keeping function	Primary care expenditure as % of current health expenditure (2020)^2^	GPs per 1,000 population (2019)^3^	GPs as % of physicians (2019)^3^	Spending on prevention as % of current health expenditure (2019)³	Hospital discharges per 100,000 population (2020)^3^
Austria	8,901,064	107.6	19.0%	58.7	81.3	Bismarck model	Single practices	No	36	1.5	28.8	2.1	20,179
Denmark	5,822,763	138.5	19.9%	58.0	81.6	Beveridge model	Group practices	Yes	55	0.8	18.8	2.2	13,598
France	67,320,216	106.3	20.5%	64.6	82.3	Mixed model	Group practices	Yes	38^4^*	1.4	44.3	1.9	16,152
Hungary	9,769,526	107.1	19.9%	62.5	75.7	Bismarck model	Single practices	Yes, partly	40	0.8	21.4	3.2	14,674
Italy	59,641,488	200.6	23.2%	68.0	82.3	Beveridge model	Single practice	Yes	n. a.	0.9	21.7	4.7	9,313

Sources: ^1^ Eurostat [134], ^2^ WHO [135], ^3^ OECD [136], ^4^ DREES [137], * referencing 2021

Researchers from Austria’s Institute for Advanced Studies (IHS) assumed the conceptualising and coordinating role in the research process. They prepared a guideline for the analysis and held several meetings with all participating researchers to ensure a uniform approach and a homogeneous analysis in all country case studies. Furthermore, the researchers from IHS were in constant exchange with the researchers from the respective countries during the entire process of data collection and analysis.

The country case studies were conducted by researchers of the following research institutes/universities: Austria – Institute for Advanced Studies (IHS), Denmark – University of Copenhagen, France – National Institute of Health and Medical Research (INSERM), Hungary – Med-Econ Human Services LTD and Italy – LUISS Guido Carli. Each researcher held a degree from social sciences and/or medicine at least at the level of a Master’s degree. Overall, 44% of the research team were female.

#### Document analysis

As preparation for the semi-structured interviews, a document analysis was carried out. It aimed to collate background information on service delivery, human resources, physical resources as well as information/guidelines/regulations provided to primary care providers. To achieve this, available literature and documents (e.g., scientific articles, grey literature, official regulations) were screened for relevant information. The results of this analysis then fed into the design of the interview guides and were used to give interviewers contextual knowledge.

#### Semi-structured interviews

Semi-structured expert interviews were conducted with different types of primary care providers, mostly general practitioners (GPs). The interviews aimed at gaining insights into the biggest challenges for primary care providers in delivering care during the pandemic as well as (suggested) measures to address them.

##### Interview guide

Using preliminary findings from the scoping review, an interview guide was developed by the IHS researcher team and piloted with several primary care providers in Austria. The interview guide was translated into the languages of the respective countries and used in all countries to enable cross-country comparisons for the case studies (see supplementary material). Researchers were free to adapt the interview guide according to specifics of their countries (e.g., cultural particularities) in consultation with the IHS research team.

##### Selection of interview partners

A purposive sample of interviewees was defined for each country by the researchers in said country and subsequently discussed with the IHS researchers. The aim was to interview at least five persons in each country and ensure sufficient variation via the following criteria: (1) at least one primary care provider from a single practice, (2) at least one primary care provider from a group practice (if applicable to the country’s health system), (3) at least one primary care provider from a rural area, (4) at least one primary care provider from an urban area, (5) primary care providers must be from different regions within the country and (6) only one interviewee per practice.

Researchers were free to invite additional interviewees in case they regarded it necessary to obtain more information. In total, 31 interviews were conducted, five in Austria, four in Denmark, five in France, six in Hungary and eleven in Italy. Summary characteristics of interview partners are presented in Table 2, a detailed description of each interview partner can be found in the table in the supplementary material.

**Table 2:** Summary characteristics of interview partners in the five country case studies

	Number of interview partners	Gender	Medical profession	Practice type	Area	Average duration (in min)
Austria	5	4 male 1 female	5 GPs	2 single practices 3 group practices	2 urban 3 rural	55
Denmark	4	2 male 2 female	4 GPs	1 single practice 3 group practices	2 urban 1 rural 1 mixed	55
France	5	2 male 3 female	3 GPs 2 nurses	4 group practices 1 other setting	2 urban 3 rural	64
Hungary	6	5 male 1 female	6 GPs	6 single practices	2 urban 4 rural	55
Italy	11	5 male 6 female	3 GPs 6 physicians in managing positions 2 nurses	1 single practice 4 group practices 6 other settings	1 urban 5 rural 5 mixed	59

Note: mixed area = area with rural and urban parts

Although the sample is relatively small, a sufficient degree of representativity was reached by deliberate selection of interview partners: In Austria, GPs were selected to represent rural-urban differences, as well as differences between regions and single vs. group practices. Two GPs have a major role in GP associations, thus channeling experiences from larger groups of GPs as well. All GPs are active in their district GP networks. In Denmark, all interviewees were/are part of quality clusters/networks of GPs that hold regular meetings. Thus, each interviewee responded with the experiences of other GPs from their network in mind. In France, the selection of interviewees was carefully conducted to achieve a geographic variation that aligns with the varying epidemiological exposure across different regions. In Hungary and Italy, special emphasis was put on selecting interviewees who are also representatives of professional associations.

##### Conducting interviews

The interviews were conducted face-to-face or via videoconference in spring and summer 2022. All interviewees were informed about the PERISCOPE project as well as about the background of the respective interviewer (e.g., educational/occupational background, research interests). Prior to the interview, each interviewee handed in a signed GDPR compliant informed consent sheet. The interviews lasted approximately 60 min and were digitally audio or video recorded. Some researchers took notes during the interviews in addition to the recordings. There was no one present during the interviews besides researchers and interviewees. Participants were informed that they could withdraw from the interview at any time. No participant payment was made to the interviewees.

##### Analysis

All interviews were transcribed verbatim from the audio/video file either by the interviewer or an independent research transcriber. The resulting transcripts were analysed using qualitative content analysis [71]. The analysis was guided by a broad category system based on the six building blocks of the WHO health systems framework (service delivery, physical resources, human resources, information and research, governance and leadership, financing). Relevant sub-categories within these broad building blocks were identified inductively for each country. The units of analysis, i.e., verbal sequences from the interviews, were coded according to the categories and sub-categories identified by the researchers using ATLAS.ti or NVivo software. The interview quotations selected to be included in the country case studies were edited into readable forms and translated into English.

### Overarching analysis

The five country case studies were subjected to an overarching analysis [72, 73] focusing on successful strategies as well as gaps and failures regarding pandemic management in primary care.

The analysis was led by the research question “What was the role of primary care systems during the COVID-19 pandemic and what can be done to increase pandemic preparedness and resilience?”. Two researchers from IHS conducted independent analyses of the themes addressed in the country case studies in order to inductively identify categories associated with pandemic preparedness and resilience of the primary care systems. Coding and interpretation of results were discussed to explore differences in interpretation of narratives, improve consistency of coding and reduce subjective influences. The categories were checked back against the country case studies by the researchers from the respective countries to ensure consistency and validity.

## Results

The main themes which emerged from the overarching analysis were grouped into the six building blocks of the WHO health systems framework: service delivery, human resources, physical resources, information and research, governance and leadership and financing. As no themes emerged in the context of financing, no results can be presented for this building block. It is important to note that the presentation of results reflects the descriptions and perceptions of interviewees, which were subjective and not necessarily exhaustive. Thus, if a theme is described for a selection of countries, this does not necessarily imply that the respective circumstance was not present in the remaining countries, but only that it did not emerge as a key theme there.

Table 3 gives an overview of the main themes that emerged from the overarching analysis. These themes represent key challenges identified by the interviewees. The following sections provide more detailed descriptions of these challenges and how they were addressed in the investigated countries.

**Table 3:** Main findings

Building block	Main themes
Service delivery	• maintaining access to and continuity of care • practical implementation of infection control measures • additional medical and non-medical tasks • compromised patient-physician relationship
Human resources	• shortage of primary care providers • increased workload of primary care providers • education and training of primary care providers
Physical resources	• lack of personal protective equipment (PPE)
Information and research	• content, timeliness and applicability of administrative guidelines • availability of up-to-date clinical information • information provision by authorities and stakeholders
Governance and leadership	• focus of pandemic response measures on hospitals • involvement in primary care policy making • weakly developed primary care system
Financing	no themes emerged

### Service delivery

A major challenge mentioned by all interviewees was shortcomings in service delivery. In all investigated countries several disruptions to service delivery were reported. Most importantly, the number of physical consultations decreased due to both supply and demand side factors. On the supply side two main issues were pointed out: closures of primary care practices and busy telephone lines in GP practices. On the demand side the most pressing issue was that patients were reluctant to enter practices because they were afraid of getting infected there, as pointed out, e.g., by an Austrian GP:

*“[…] many patients no longer dared to go to the GP practice because they were afraid of an infection. In the beginning, it was presented very dramatically. Especially when we saw the pictures from Italy, where rows and rows of coffins were driven around. Of course, that created a great deal of fear. And that also kept many patients from coming to the GP practice.” – AUT_IP1*.

Hence, it was pivotal for primary care providers to sustain service delivery and overcome the reluctance of patients, while at the same time ensuring a certain level of safety through physical distancing. Four sub-themes were identified in this context: (1) maintaining access to and continuity of care, (2) practical implementation of infection control measures, (3) additional medical and non-medical tasks and (4) compromised patient-physician relationship.

#### Maintaining access to and continuity of care

In all investigated countries primary care providers put measures in place to ensure access to and continuity of care. The most prominent measure was the establishment of telehealth services (e.g., teleconsultation via phone or video conference platforms). Further measures included triage and home visits.

In all investigated countries the interviewees agreed that teleconsultations were valuable in providing care to patients, especially in times when physical distancing was of importance. However, interviewees also identified various limitations of teleconsultations. In particular, elderly patients were observed as having difficulties with the shift to teleconsultations. Moreover, potentially overlooked diagnoses and insufficient IT equipment for video consultations were mentioned. For instance, a French primary care provider missed the opportunity to conduct physical examinations and a Hungarian GP emphasised the importance of the primary care clinic as a social hub for elderly patients:

*“We had to do teleconsultation. Now, that said, with teleconsultation we cannot palpate patients, we do not have clear clinical observation, things can be missed. We have realised this, so there were barriers to the use of teleconsultation.” – FRA_IP5*.

*“One of the vulnerable groups are elderly people living alone […] who want the clinic to open already, because it is a meeting point for them, an important meeting point that ceased to exist in their lives during the epidemic wave, and they became isolated.” – HUN_IP3*.

Furthermore, interviewees pointed out the importance of improving digital literacy for elderly patients. They viewed telehealth as conducive to promoting patient empowerment as described by Italian GPs:

*“For patients over 70, despite not being accustomed to the use of technological tools, we have made a training, […] which has proved effective, precisely to overcome digital illiteracy.” – ITA_IP7*.

*“Managing patients at home means making them feel isolated but not alone, also through communication: they knew when to call the family doctor, […], they knew that a contact-tracing center would call them to find out about their health.” – ITA_IP3*.

Apart from telehealth, interviewees reported several other measures to ensure access to and continuity of care. These include (pre-)triage, adjusted home visits or primary care providers actively informing their patients about infection control measures in their practice (e.g., using PPE and regular testing of the staff). The primary purpose of (pre-)triage and home visits varied between the respective countries. While triage in Italy focused on efficiently managing the accumulated backlog of postponed appointments (e.g., avoiding unnecessary consultations), the response in Austria and Hungary was concentrated on the coordination of care of potentially infectious patients:

*“We asked those who have these symptoms to call first, and then we discuss when they come or whether they should come to the clinic at all, so we coordinated the care.” – HUN_IP1*.

Regarding home visits, two different approaches were described. Both prioritised the safety of patients in reducing their exposure to potentially infectious persons. Austrian and French interviewees aimed at maintaining access and continuity of care and thus intensified the number of home visits. Italian interviewees, on the other hand, aimed at the safety of vulnerable patients from infections and thus decreased the number of home visits as described by a GP:

*“COVID has impacted the visits at home for the patients with more needs. We think of heart failure, heart patients, diabetics, which we family doctors used to see often. These visits were reduced, because there was concern, not so much for the professional, but for the patient.” – ITA_IP9*.

Despite efforts of providers to maintain access to and continuity of care, they noted that some patient groups were particularly affected by disruptions to service delivery in primary care and consequences of physical distancing measures. In particular, this pertains to vulnerable patients such as chronically ill and mentally ill persons, elderly persons, as well as children and adolescents. In Italy, for example, clinics were established in remote areas to provide care to chronically ill patients unable to access specialist care. These clinics were managed by nurses and aimed to ensure continuity of care and avoid hospitalisations. In Denmark, physicians expressed concerns about underprovision of care to children and adolescents during the pandemic. Thus, they encouraged young patients to reach out to their peers to make sure that they seek care, when necessary, as described by a physician:

*“It was especially the young people, after a year or so. Then I started being proactive about young people and where they were. […] And now I can see that there is this wave in child and adolescent psychiatry, those are all the severe cases, but there are also the moderate cases.” – DNK_IP1*.

*”If I saw a young person in the clinic, I often said to them, ‘you should let your friends and social circle know that they should contact their GP if they have well-being-related problems or they should articulate it at school’.” – DNK_IP1*.

Challenges were also reported regarding referrals to either hospitals or specialists. Specifically, difficulties emerged due to inappropriate guidelines, overflowing hospitals, or the redeployment of specialists to COVID-19 care. Interviewees in Austria and Denmark, explicitly mentioned that early public communication or official guidelines were perceived as harmful as they suggested that patients – those with COVID-19 symptoms, but also any other patients – were only to be treated in hospitals. Primary care providers thus felt they were not put to use in the most efficient way and should have been more involved in the pandemic response instead of exacerbating the situation in the hospitals through unnecessary referrals:

*“Because we usually weed out nine in ten patients by seeing them in the clinic, or even more. So we have also sent way more people to be examined in hospitals based on the circumstances at the time. Because our guidelines said that they should not show up at our clinic.” – DNK_IP3*.

Nonetheless, primary care providers were urged by necessity to soften the impact of overflowing hospitals as these were unable to admit further patients. In particular, some COVID-19 patients who would normally have been hospitalised were only monitored at home by the GP, raising questions of compromised care as remarked by a French physician:

*“During the first wave, of course, I also had patients who were unwell, whom I wanted to hospitalise. So I called 15 [emergency phone number]. The doctor on the 15 [said] that it wasn’t even worth thinking about, that the emergency unit was overflowing in every direction, that my patient with such advanced age, would be sent home anyway. So we offered poorer quality care. We put people on oxygen at home in the hope that they would survive.” – FRA_IP3*.

Moreover, given hospital capacity constraints primary care providers faced difficulties in assessing which patients were most in need of hospitalisation. However, video consultations rather than phone consultations were reported as a remedy, for example, by a Danish physician:

*”The video helped. Definitely. I think so. We caught some people that way. You could see in the image how badly affected they were. And then you could hospitalise them based on that.” – DNK_IP1*.

In Austria and Hungary, interviewees also reported difficulties in referring patients to specialists. Austrian interviewees suggested that apart from strengthening pre-existing integration of primary and secondary care, teleconsultations should also be possible among providers so GPs could consult with specialists without the need to present patients in person. Dedicated time slots provided by specialists for such consultations would be needed for such an approach.

#### Practical implementation of infection control measures

While it was important to reduce congestion in practices through teleconsultations, (pre-)triage and home visits, some consultations could only be delivered in-person at the practice. Thus, it was essential to implement infection control measures to limit the spread of the virus in primary care practices. In this context various measures were put in place in all countries. For example, there were reports of primary care providers using separate treatment rooms for COVID-19 and non-COVID-19 patients in France, flexibly utilising the space outside or inside the primary care practice (e.g., examining patients at the parking lot in Denmark, providing outside benches for safe waiting in Hungary, using separate entrances in Austria) and installed air purifiers in Hungary. If separate entrances into the practice were logistically unfeasible, dedicated contact hours for potentially infectious patients were used in Austria. Moreover, emphasis was put on regular testing of staff in Denmark. Although various on-site infection control measures were mentioned, the flexible use of rooms emerged as most pivotal in ensuring a safe practice environment as pointed out by an Italian physician:

*“I would absolutely like to emphasize that it was strategic – and in my opinion will continue to be strategic – to be able to manage the spaces. It seems trivial, but we suddenly found ourselves having to completely rethink the logistics of our rooms, adapting them to new functions.” – ITA_IP5*.

Nevertheless, a Danish physician also considered the limits of on-site infection control measures when dealing with an airborne disease:

*“It is difficult when it is airborne. Then there are limits. We have the rooms that we have here, right? […] we cannot disinfect and clean all over. And yes, we could ventilate more and stuff like that, but then we could only see patients one at a time, maybe one per hour, if we were to do all these things. So it sets some limitations when things are the way they are with airborne infections.” – DNK_IP1*.

#### Additional medical and non-medical tasks

The pandemic also brought about additional tasks for primary care providers, often without additional capacities, as specifically emphasised by French and Hungarian interviewees. These new responsibilities included: medical care such as involvement in COVID-19 testing or covering for absent specialists, but also non-medical care such as organising food deliveries for homebound patients, helping with the online registration for the vaccination appointment or increased administrative responsibilities. A Hungarian GP was frustrated by the administrative burdens associated with, among others, testing and vaccinating:

*“Why do I have to make an appointment for the patient’s vaccination? Why should I make an appointment for the rapid test? We have six phone lines, but only two ears. Dealing with this was very, very difficult. At the end of the day, 30–40 e-mail drug requests had to be answered. The administrative burdens were terribly high.” – HUN_IP5*.

A French physician described how they organised food deliveries to elderly patients but also dealt with toothaches:

*“I’ve been supplying food to elderly patients because professional carers were not doing home visits anymore, so they had nothing to eat and the neighbours weren’t interested in helping them.” – FRA_IP1*.

*“We had to deal with [patients of] my dear fellow dentist and ophthalmologist who had left without even giving a telephone number. So we dealt with raging toothaches…“ – FRA_IP1*.

These examples not only illustrate the frontline role of GPs but also the necessity to integrate primary care practices within the community.

#### Compromised patient-physician relationship

The pandemic also affected the patient-physician relationship due to manifold factors. In Denmark, the lack of contact with vulnerable patients was said to have negatively impacted the patient-physician relationship:

*“Our relational work has suffered during the COVID pandemic, right? […] I have been a GP for 25 years and I know a lot of my patients well. Ups and downs. But you cannot compare, it is not the same intensity […] So that care, which is also a significant part of our work, we have not been able to provide that.” – DNK_IP2*.

Most notably, in Austria and France, primary care providers reported to have experienced aggressiveness from patients and observed the spread of misinformation via social media and mass media negatively impacting the patient-physician relationship. Hence, it was seen as imperative to take measures to improve health literacy of the population to allow for a more adequate appraisal of information:

*“[…] people have simply become increasingly grumpy. The mood has even been affected here in the countryside. We have lots of super nice patients, but that has really changed in the course of the pandemic. So, like everywhere, the toxicity has become commonplace, in part it was somehow no longer bearable and the assistants in the waiting rooms have already faced real problems, whereas it occurred much rarer for the colleagues […] The assistants, who, by the way, are constantly forgotten, yes, our assistants, they have really performed.” – AUT_IP4*.

*“I think that it has greatly degraded our relationship with the patient. […] we have people who, it happens much more regularly now: people come with scientific articles, but which are of lesser quality or whatever, well, that they don’t really understand and they are quite vindictive.” – FRA_IP4*.

Furthermore, Austrian GPs reported misinformation as well as difficulties to win persons over for vaccination. Hungarian GPs faced negative attitudes from patients towards COVID-19-related measures (e.g., refusal to test for symptoms suggestive of COVID-19, refusal of hospital treatment, non-compliance with quarantine rules, refusal to vaccinate).

### Human resources

Three major problems were reported by the interviewees in context of human resources: (1) shortage of primary care providers, (2) increased workload and (3) insufficient education and training. A shortage of primary care staff was explicitly pointed out by interviewees in France, Hungary and Italy. While this was already the case before the pandemic, the adverse working conditions of the two pandemic years have exacerbated the situation in inducing many providers to quit the profession.

Furthermore, primary care providers in Austria, France and Hungary specifically mentioned an increased workload. An Austrian GP, for example, described difficulties in providing care for a large number of patients at home:

*“It has to be said that there are many patients in quarantine, up to 1,800, up to 2,000 patients per quarter, so it was a lot of work, and of course with home visits and telephoning it was an increase in workload. […] But that was providing care for patients at home, who have acute problems and acutely need a phone call and you struggle with this large number of patients. But this is urgent, and meeting these requirements, that was a challenge.” – AUT_IP5*.

The pandemic also changed the nature of work for primary care providers. Thus, it was also necessary to educate and train personnel for the adjusted requirements. In Austria, interviewees emphasised that specific training, quality circles and interpersonal networks would help to improve resilience. In Hungary, interviewees suggested that primary care staff (i.e., physicians and nurses) should be trained in the basics of COVID-19 and hospital care during calmer periods between waves. This would allow a more flexible personnel deployment if the need arises.

### Physical resources

In all countries, a major challenge raised by interviewees in the context of physical resources was the lack of personal protective equipment (PPE). This was especially true at the beginning of the pandemic. Insufficient supplies of masks, protective suits and hand sanitisers endangered primary care providers with some noting the higher priority given to hospitals, as in Denmark for example:

*“We did not have enough protective equipment in the clinic to be able to see all patients wearing coveralls and masks, etc. These things were not delivered to us to the same extent as to the hospitals.“ – DNK_IP3*.

Furthermore, Hungarian interviewees described the poor quality of imported personal protective equipment as a hindering factor.

Measures to deal with the shortage of personal protective equipment were explicitly mentioned only by Austrian and French interviewees. They reported that primary care providers issued public calls for donations of PPE, shared instructions on how to refurbish used masks, asked local artisans or physiotherapist friends for masks and used snorkel masks or masks from remaining stocks of a previous pandemic:

*“Well, we were stressed a lot… We were stressed because we simply didn’t have [PPE] […] We asked local artisans to give us protective masks. I took out the masks I have kept from the avian flu pandemic.” – FRA_IP2*.

Interviewees in France and Hungary pointed out that global supply chains create dependencies and suggested, as a countermeasure, that Europe should have the capacity to produce medical supplies and personal protective equipment:

*“There were many phases where we lacked something. And often, we lacked things because it was impossible to produce [medical supplies and PPE] on the national territory. […] When everything is disorganised, when we are in a situation of crisis, we must be able, I think, to secure the essentials of life to the community.” – FRA_IP3*.

### Information and research

Three major challenges were described by the interviewees in the context of information and research: (1) content, timeliness and applicability of administrative guidelines (e.g., for isolation or quarantine measures), (2) availability of up-to-date clinical information and (3) information provision by regional and local authorities as well as stakeholders.

#### Content, timeliness and applicability of administrative guidelines

During the pandemic, a lot of guidelines by public authorities were issued, e.g., regarding procedures on how to handle or register COVID-19-positive patients or when and how long to quarantine. Interviewees from all investigated countries criticised either the content of the respective administrative guidelines, their timeliness or their applicability. For example, it was criticised that guidelines were communicated vaguely in press conferences, and generally published too late, on short notice or without approval by the responsible authority. A Danish primary care provider was frustrated with short-notice publications of guidelines as it left them with insufficient time to implement and familiarise themselves with the guidelines. They felt that this was detrimental to their perceived trustworthiness:

*“They [the new guidelines, ed.] spark a lot of activity with us and then we are not equipped […] that is experienced as extremely unprofessional. I mean, it puts us in a negative light. […] it is not conducive to trust from our patients that we do not know things that they have been able to read on some website or Facebook or have heard on the radio news or something […] The problem was that decisions were made outside of our organisation and frameworks. Which were announced to the public and that is great. But we should have just had them in advance, maybe just a few hours or the day before, when they were decided on.” – DNK_IP2*.

With regards to contents of the administrative guidelines, primary care providers felt that guidelines were confusing, updated too often, lacking vision or actively harmful. Specifically, Hungarian and Italian interviewees mentioned the incomprehensibility of guidelines. Moreover, primary care providers in France and Italy were particularly disappointed by the lack of a recommendation in favour of mask wearing:

*“At the beginning, they told us that masks were not necessary. And then, yes wearing a mask was required. So no, at the very beginning, it was really… yes, a lot of nonsense. We have to be honest […] But it’s true that no, even at the beginning wearing FFP2 [N95] masks, that wasn’t what was recommended.” – FRA_IP2*.

*“At the beginning internally, we were told not to wear masks so as not to scare the patients and so honestly this was quite a shocking thing. At the beginning, however, no one knew anything, no one understood, then we realised that instead the masks were perhaps quite useful.” – ITA_IP1*.

The applicability of administrative guidelines was criticised by interviewees in Austria, Denmark, Italy and Hungary. In Austria, the heterogeneity of guidelines/regulations between different states or even districts made a unified response difficult. In Denmark, guidelines differed between types of health care providers which led to challenges for practices co-located with other kinds of providers, e.g., GPs and physiotherapists. In Italy and Hungary, guidelines were perceived as too generic and difficult to adapt to local conditions as described by a Hungarian GP:

*“Regarding the guidelines, which come from Budapest, it was not necessarily entirely good for us. It had to be translated to local conditions.” – HUN_IP6*.

#### Availability of up-to-date clinical information

In the emerging pandemic, clinical information on transmission, contagiousness, usefulness of tests, treatment regimens and predictors of severe disease progression was continuously produced but spread across preprints and conference proceedings. Thus, it was difficult for each primary care provider to efficiently collate all emerging information. In Hungary, e.g., a GP association used their Facebook page to spread information among colleagues. Austrian interviewees praised the development of a unified online platform by the Austrian Association of General Practice (ÖGAM) together with the Karl-Landsteiner Medical University. This platform provided the latest evidence and practical tips together with a helpdesk, newsletter and podcast, as described by one of the initiators:

*“And we tried to provide practice organisation with process descriptions and diagrams […] and on the first day we were online, that was March 22 2020, we had 15,000 visits within 24 hours.” – AUT_IP4*.

#### Information provision by regional/local authorities and stakeholders

Generally, interviewees from all investigated countries named two main problems regarding information provision by regional and local authorities as well as stakeholders: lack of clear information for both patients and primary care providers and the substantial amount of misinformation and fake news circulating in the media.

On the patient side, information from the government or its instituted bodies was considered contradictory and lacked elements of public health communication like instructions for appropriate mask wearing. Relatedly, interviewees felt patients were reducing their primary care visits due to falsely assuming that primary care practices had closed, patients not wanting to “bother” their GP, and the government instilling fear in patients and essentially communicating not to visit primary care practices.

Interviewees mentioned several measures to alleviate the negative impacts of poor information provision from authorities. To improve knowledge of patients, for example, Hungarian primary care providers displayed information in windows of the practice or initiated personal “press releases” on local radio, TV, newspapers or social media. In Denmark, a national information campaign was launched to inform the population of the availability of GPs and the importance of seeking care when necessary. Additionally, primary care providers spent considerable time to provide COVID-19-related guidance to patients who were uncertain of how to interpret official guidelines and information as mentioned, for example, by a Danish physician:

*“We just spent a lot of time handling corona, all kinds of questions from patients who could not find proper guidance on websites and who would ask us out of frustration [...].” – DNK_IP4*.

On the provider side, Hungarian interviewees felt they did not receive enough information early in the pandemic but noted improvements over time, as reported by a GP:

*“Several times colleagues felt that they were left without information for a long time. I felt that in the middle of the crisis, the public administration slowly picked up the rhythm.” – HUN_IP3*.

In France, interviewees were frustrated that the government tended to share information first with the media before communicating with primary care providers. In the case of the temporary withdrawal of the AstraZeneca vaccine, this even put them in legal danger, as described by a physician:

*“[National] authorities always communicated first to journalists before communicating to health professionals, which has put us in an extremely difficult position. […] we learn, I learn from patients at around 3pm, I think it was, so I don’t remember if it was 3pm or 5pm, that the vaccine is withdrawn from the market […]. And we [the GPs] don’t get the email until 9pm. I think it’s intolerable that FranceInfo [FranceInfo is public 24-hour cable news channel] get the information before us, because we are prescribers. In other words, it even put us in legal danger.” – FRA_IP4*.

As the shortcomings in information provision also affected primary care providers, they decided to hold internal meetings and relied on their own experiences and information gathered from formal or informal networks of colleagues. Professional networks appeared to be particularly valuable for primary care providers as they hosted Zoom sessions to discuss relevant issues, such as vaccinations:

*“That gave us arguments to use on patients. They explained to us how vaccines work. And right away, even before the vaccination centres opened. So in fact we already had the elements, it gave us knowledge and tips to provide answers [to our patients].” – FRA_IP5*.

Another issue reported by interviewees of all countries except Denmark was the prevalence of misinformation or fake news. They emphasised the spread of misinformation via social media, in particular Facebook, but also mentioned the harmful influence of mass media in propagating unscientific notions and stoking radicalism. Primary care providers felt the spread of misinformation damaged patient-physician relationships, led to unjustified opposition from patients and caused confusion in the population. This also made vaccination efforts more difficult. A Hungarian GP pointed out how misinformation contributed to uncooperative behaviour of substantial parts of the population:

*“A significant part of the population had access to much more negative and false information on Facebook, which led to their unwarranted fears and unwarranted resistance.” – HUN_IP3*.

As a way to resolve or at least improve the effects of misinformation and fake news, the importance of government action to counter the flow of misinformation, health literacy and face-to-face communication were highlighted:

*“We need a different kind of communication with citizens, because the other terrible aspect has been the instrumentalisation of people on social media, this radicalisation into totally unscientific positions. […] Communication with citizens is not done by posters: it is done face-to-face with people [...].” – ITA_IP4*.

*“Patient education, I feel that it could be a great step forward, because we can also help our own work with it. Patients should have been better informed so that there were no misunderstandings.” – HUN_IP6*.

### Governance and leadership

A major challenge and learning for the future was that effective governance and leadership of health systems is necessary to adequately respond to crisis situations such as the COVID-19 pandemic. This fact was mentioned in a lot of interviews, and was particularly evident in Austria, France, Hungary and Italy.

With regards to governance and leadership, interviewees mentioned various weaknesses with some similarities between countries. The most striking challenge was that pandemic response measures mainly focused on hospitals with primary care being largely ignored, as, for example, reported by an Austrian GP:

*“Yes, so at the beginning of the pandemic, somewhat surprising for me, […] was the fact that primary care was not supposed to play a role in the pandemic. […] What was really hard to observe was that primary care does not actually appear in any planning in the health care system. […] The hospitals always appear somewhere and then immediately have roles and it is immediately defined. There are tents built, triage systems made, […]. Only for primary care there is no plan.” – AUT_IP2*.

Danish primary care providers would have preferred to play a greater role in the pandemic response, in particular by relieving overburdened hospitals from providing care to patients that could also have been treated in primary care. Furthermore, they were concerned about the low level of involvement in primary care policy making. A physician stated that guidelines could have been clearer if GPs had played a greater role in developing them:

*“Like I also described before – that guidelines for us and for the patients were more clinical. General practitioners need to be involved in developing such things. And not too much rubbish or too useless for the patients and definitely also for us.” – DNK_IP4*.

In several countries interviewees reported on inefficient cooperation between primary care providers and authorities. In Austria, for example, primary care providers experienced difficulties in working with local authorities, often waiting in vain for answers. In Hungary, the cooperation between primary care providers and authorities was reported as person-dependent and not well-regulated resulting in delayed decision-making and non-constructive communication. In Denmark, the need to strengthen cooperation between general practice and municipal health services was pointed out:

*”The cooperation within the municipalities with us has become more important. And maybe we are lacking a body to support this. […] I just saw it very clearly, that a lot of the management of the pandemic happened only in the hospitals. Whereas we are actually fighting the pandemic just as much in the municipalities and amongst ourselves. So I believe there is a need to think, unfortunately, about the organisation of the health system.” – DNK_IP4*.

Furthermore, Italian and Hungarian interviewees identified pre-existing weaknesses of the primary care system which were exacerbated during the pandemic. In Italy, the fragmented care delivery system and regional disparities hampered a standardised response to the pandemic. In Hungary, traditional operating structures of GPs^1^ could not meet the increased workload of administration and patient care at the same time. Moreover, a GP bemoaned the weak public health system in Hungary:

*“[…] public health tasks cannot be put on primary care. In fact, it has now been proven that there is no organised, mobilised public health system in Hungary.” – HUN_IP5*.

## Discussion

Our analysis provides insights into the role of primary care in five European countries over the course of the pandemic. As our country selection aimed at broad variation in terms of population and health system characteristics, we expected larger differences between countries’ learnings. We expected differences resulting from the type of health care system, the predominant primary care model or the strength of the primary care sector in the respective country. However, themes emerging from the overarching analysis unveiled striking similarities, but hardly any differences across the five analysed countries. There was a common notion among interviewees that primary care was not the priority of policy makers and instead sidelined by a focus on secondary care. One could have expected that countries with a strong primary care system would utilise and adequately incorporate primary care into their pandemic response. However, interviewees in countries with relatively weak (e.g., Austria) as well as relatively strong (e.g., Denmark) primary care systems reported similar tendencies of feeling overlooked and not sufficiently included into pandemic management. Furthermore, disruptions to service delivery were experienced by interviewees in all countries. These were mainly caused by patients’ fear of contracting the virus in GP practices, which was driven by dramatic footage from Italy in the beginning of the pandemic. Similarly, the provision of guidelines was criticised in all countries, but some differences emerged in this regard. While interviewees in more centralised countries, like Hungary, perceived guidelines as too generic and not adapted to local conditions, those in more decentralised countries, like Austria, felt that heterogeneity of guidelines hampered a unified crisis response.

We conclude from our analysis that the major challenges faced by primary care providers – especially in the wake of the pandemic – were broadly similar across quite different European countries. Learnings from these common experiences can thus be of common value to providers and policy makers in several countries who aim to foster preparedness of the primary care sector for future disruptive events.

In the following, we provide learnings from the overarching analysis along the building blocks of the WHO health systems framework. Based on insights from the interviews, we also suggest measures to improve preparedness for future pandemics and to increase resilience of primary care systems. Applying the lessons learnt from the pandemic should not only make primary care systems more resilient against future pandemics but may also help stem the burden from other disruptive events such as antimicrobial resistance, armed conflict, climate change, environmental disasters or social unrest. For example, many disruptive events provoke mobility restrictions, due to real obstacles (e.g., flooding) or due to precautions and danger (e.g., social unrest). Thus, a functional primary care (infra)structure becomes even more crucial in terms of accessibility under unstable conditions. Concentration tendencies in the hospital sector in several countries underline this geographical reasoning even further. Furthermore, increased telehealth proficiency may be valuable in such cases. When transferring learnings from the COVID-19 experience to more general disruptive events, lessons need to be adapted or generalised – which has happened to some degree already. The learning that PPE stocks need to exceed the average use to ensure preparedness in unforeseen circumstances can be transferred to other supplies that might become relevant in disruptive events, such as material for dressing wounds. The understanding that proper hand hygiene and wearing masks in populated spaces can lower infection rates and possibly lower mortality can be applied in the recurring flu season.

### Service delivery

Access to and continuity of care are among the basic determinants of quality of primary care [74, 75]. Maintaining access and continuity is particularly challenging during health crises. Disruptions to regular service delivery in primary care during the COVID-19 pandemic have been widely discussed in the literature [76–78] and are especially relevant for vulnerable patient groups such as patients with chronic diseases [47, 79] or elderly patients [36, 37]. Such disruptions were also reported by the interviewees of all investigated countries. The primary care providers interviewed for our analysis put measures in place to ensure access to and continuity of care. The most prominent measure was the establishment of telehealth, which turned out to be an asset for reducing disruption of care. However, in many countries the implementation was quite *ad-hoc*. Thus, issues on available software, financing and data protection were not always clear. In order to increase pandemic preparedness, the implementation process should be streamlined and the mentioned issues should be resolved in advance. Furthermore, telehealth would have been even more effective if primary care providers had been more experienced in its use. With telehealth, it is crucial that providers understand in what cases the benefits of telehealth outweigh its drawbacks and, thus, which mode of care delivery is preferable [80–82]. Home visits, on the other side of the spectrum, are necessary, especially, with persons in isolation, quarantine or in ill condition. However, in some of the investigated countries, home visits ceased due to the fear of infection. In order to increase pandemic preparedness in this case, dedicated teams doing home visits might be a solution. In rural areas, this could be organised by local GPs in a kind of duty roster.

Primary care has a central role in coordinating care within a health system [83]. Therefore, it is imperative to ensure a coordinated care delivery during health crises, e.g., to prevent the overburdening in hospitals [84]. The COVID-19 pandemic has disrupted coordinated care delivery as well as traditional workflows [85–88]. Such disruptions were also identified by interviewees of several investigated countries, especially between primary and secondary care. Given these problems, primary care providers in several investigated countries monitored COVID-19 patients at home who would normally have been hospitalised or dealt with conditions that would normally have required specialist care. In order to increase pandemic preparedness in this context, GPs should have the opportunity to either refer patients to hospitals for physical consultation or seek assistance from specialists via provider-to-provider telehealth. Furthermore, strengthening primary care and its coordination, especially with secondary care, would be beneficial for everyday care, and at the same time ensure that patients continue to receive required services during a crisis.

Another important aspect during health crises such as the COVID-19 pandemic is the implementation of infection prevention and control measures. Such measures are of critical importance in maintaining service delivery and protecting vulnerable patient groups [89, 90]. A variety of such measures for primary care have been proposed in the literature [5, 23, 91–94]. The primary care providers interviewed for our analysis described several measures from their own practice. The most prominent were measures enabling physical distancing, e.g., examining patients in the parking lot, providing outside benches for safe waiting or using separate entrances to the primary care practice. To increase pandemic preparedness in this respect, primary care practices need to improve their quality in terms of process and structure. They could, for example, use mandatory booking systems for medical appointments to space out patients instead of having overcrowded waiting rooms. Patients without a booked appointment could be necessarily triaged with a checklist for likeliness of infection and urgency of unscheduled consultation. Furthermore, dedicated opening hours for infectious patients could be implemented. Additionally, measures should be taken to improve air quality in waiting rooms. Where possible, newly built primary care practices should have separate entrances for infectious patients.

### Human resources

Qualified and motivated health professionals are a prerequisite for high-quality care delivery in “normal” times, and probably even more so during health crises [95]. Primary care can relieve hospitals in crisis situations, as illustrated during the H1N1 influenza outbreak in 2009 [96–98]. However, during the COVID-19 pandemic support measures were more focused on hospital staff than on primary care staff [12, 99–102]. Accordingly, the interviewees in all investigated countries reported not being recognised to the same extent as their colleagues in hospitals. This was also true for countries with strong primary care systems such as Denmark. To increase pandemic preparedness in this respect, primary care providers should be more involved in the pandemic response [9, 103], e.g., receiving sufficient PPE in time to allow for safe care delivery, but also by including their professional representatives in crisis management committees.

Furthermore, dynamic efficiency in health systems requires sufficient human resources without everyone working at their physical and mental limit during “normal” times. Already before the pandemic, staff shortages were reported in primary care with professionals suffering from high levels of stress and burnout [104–106]. A high prevalence of burnout and dissatisfaction not only threatens the wellbeing of health professionals themselves but may also negatively impact patient experience, population health and costs [107]. During the pandemic, many articles and surveys reported an increase in burnout, stress and depression rates among health professionals including primary care providers [22, 108–110] although some also noted stable [111] or even declining rates [112]. The adverse working conditions including high workload were seen as exacerbating the situation even further [60, 113] with interviewees of several investigated countries agreeing with that notion. Likewise, the labour market situation deteriorated with the health sector being particularly affected by staff shortages [62, 114]. Therefore, to increase preparedness for future pandemics, more emphasis should be put on improved working conditions in order to recruit and adequately train a sufficient number of primary care staff, i.e., GPs, nurses and practice assistants.

Moreover, several articles and surveys report feelings of unpreparedness among primary care providers [9, 93, 115, 116], and this is also echoed by several interviewees in our analysis. Lamberti-Castronuovo et al. [84] appraised the literature and found substantial support for training at the local level, and identified four relevant skill groups for training curricula: (1) basic skills and concepts of disaster management (e.g., office preparedness, human resources management), (2) clinical and technical skills (e.g., triage, counselling and psychological aid), (3) public health emergency skills (e.g., prevention control standards, risk communication, use of PPE) and (4) additional skills (e.g., teamwork, internal communications). The primary care providers interviewed for our analysis suggested establishing quality circles, offering specific training and exploiting interpersonal networks to tackle a lack of preparedness. Additionally, interviewees thought enabling a more flexible deployment of medical staff in primary care and hospitals through suitable educational measures would have contributed to a more robust health system. Indeed, throughout the pandemic several instances of redeployment were documented [18, 99, 117, 118]. Although the staff reported both positive and negative experiences [18], redeployments were seen as instrumental in meeting increased demand [118].

### Physical resources

Physical resources in a primary care context encompass access to capital infrastructure (e.g., physical capacities, digital technologies) and medical resources (e.g., PPE). Interviewees in all investigated countries criticised a lack of PPE, especially in the early periods of the COVID-19 pandemic. Shortcomings in this respect have been broadly discussed in the literature [59, 117, 119–121]. Sufficient availability of PPE is particularly important as it protects both providers and patients [122, 123]. Moreover, healthcare professionals without adequate access to PPE have been shown to be at greater risk of suffering from mental health problems than those with adequate access to PPE [124]. In some cases, practices also closed because of a lack of PPE [125]. Possible measures for enhancing availability of PPE, and thus improving pandemic preparedness include ensuring regional production capacities and reducing dependencies on global supply chains.

### Information and research

Timely provision of valid information is essential to guide an effective response to health crises [126]. The content, timeliness, availability and applicability of guidelines have been reported as problematic during the COVID-19 pandemic, both in the literature [117] and by the interviewees of several investigated countries. According to the interviewees, primary care providers would have preferred clear guidelines from the beginning, even if they needed adaptions as new evidence continued to emerge. To be better prepared for future pandemics, clear and applicable guidelines not only for GPs but also for other health professions like nurses, social workers, rehabilitation therapists, pharmacists, nutrition specialists and psychologists would be needed. These should then be made widely available, e.g., through a dedicated server that everyone has access to, so they can retrieve the information whenever needed. Decision-makers should, in turn, have access to up-to-date data from the primary care sector (e.g., closed practices or adapted opening hours, availability of staff and PPE in practices) to allow them to adapt guidelines and measures to the current situation.

Furthermore, primary care providers encountered (deliberate) spread of misinformation during the COVID-19 pandemic. This issue has been reported in the literature [127] as well as by many interviewees in our analysis. To increase pandemic preparedness in this respect, governments and media need to ensure that information is accurate and communicated in a target-group specific way so that patients can confidently follow evidence-based information. For many governments, this may mean that they need to be more aware of the importance of communication in pandemic situations and build capacities accordingly. Improved communication capabilities might also help with other issues like vaccine hesitancy or healthy diet during “normal” times, and lay the foundation for resilience against misinformation, i.e., health literacy.

### Governance and leadership

For the primary care sector to respond timely and effectively to an emerging health crisis, clear policies regarding functions and roles of primary care in such crises would be required. However, such policies are often lacking or are underdeveloped [9, 84]. To be prepared for health shocks, the literature suggests the creation of a comprehensive preparedness plan which integrates all areas of a health system, namely public health, primary care, secondary care as well as long-term care [84, 128–131].

Moreover, structural shortcomings in the context of policy planning during the COVID-19 pandemic have been discussed in the literature. These included, e.g., an insufficient pandemic preparedness [9] and a fragmented health system resulting in criticalities in patient care [101]. Additionally, the response to the pandemic was criticised in its lack of involvement of primary care providers in decision making and planning of processes [103, 132, 133] and its neglection of primary care providers by health authorities [113, 119, 133]. Our analysis supports this view and gives examples of what could have worked more smoothly. In order to increase pandemic preparedness, detailed pandemic management plans should be developed. From the perspective of primary care providers, clear leadership and guidance would be necessary to be better prepared for future pandemics. This includes, for example, dedicated process owners that make decisions for their area of responsibility, standardised regulations for primary care and a well-working interface to authorities, as well as applicable data protection regulations to enable smooth data exchange between primary care providers and authorities.

## Conclusion

The COVID-19 pandemic has demonstrated the importance of health crisis preparedness and the resilience of health systems. Both concepts have been the subject of health policy research long before the outbreak of the COVID-19 pandemic, partly induced by previous health crises. Nevertheless, many health systems were not well prepared for the COVID-19 pandemic.

A key learning from our overarching analysis is that the potential of the primary care sector to contribute to pandemic management was not sufficiently used during the COVID-19 pandemic. Governments focused their attention on the hospital sector to avoid a situation in which COVID-19 patients would die due to lack of capacities. Nevertheless, primary care could have helped easing the pressure on hospitals while minimising the negative indirect health effects for non-COVID-19 patients.

Furthermore, our analysis found that although the primary care sector was not well prepared for a pandemic, it adapted surprisingly well as the pandemic progressed.

Finally, we conclude from our analysis that a good primary care system can only be achieved if a strong integration with secondary care exists, ultimately resulting in greater resilience in case of a shock or health crisis. This integration of systems will also mean that health structures can provide high-quality care during “normal” times.

To the best of our knowledge, this is the first in-depth cross-country qualitative analysis of lessons learnt from and for the primary care sector during the pandemic. Thus, it serves as a valuable source for improving preparedness and can support health systems in performing at their full potential in future pandemics.

### Electronic supplementary material

Below is the link to the electronic supplementary material.

Supplementary Material 1: Interview guide and individual characteristics of interview partners in the five country case studies

## Data Availability

The datasets used and/or analysed during the current article are available from the corresponding author on reasonable request.

## Footnotes

1 The traditional operating structure consisting of the GP and a nurse was unable to cope with the increased medical and administrative workload associated with the pandemic.

## Abbreviations

AUT: Austria
COVID-19: Coronavirus disease 2019
DNK: Denmark
FRA: France
GDPR: General Data Protection Regulation
GP: General practitioner
HUN: Hungary
IHS: Institute for Advanced Studies
IP: interview partner
ITA: Italy
PERISCOPE: Pan-European Response to the ImpactS of COVID-19 and future Pandemics and Epidemics
PPE: personal protective equipment
