# Supplementary material for: The role of primary care during the pandemic: shared experiences from providers in five European countries
Source: BMC Health Serv Res. 2023 Oct 2;23:1054. doi: 10.1186/s12913-023-09998-0 (PMC10546726; doi:10.1186/s12913-023-09998-0)
Supplement: Supplementary file 1 — Supplementary Material 1: Interview guide and individual characteristics of interview partners in the five country case studies [file 12913_2023_9998_MOESM1_ESM.docx]

**Supplementary material**

1. Interview guide
2. Table: Individual characteristics of interview partners in the five country case studies

Interview guide

| Interview ID | |  | |
| --- | --- | --- | --- |
| Name/affiliation/position of interviewee(s) | |  | |
| Practice type | | O single practice O group practice O other: _______________________ | |
| Practice location | | O urban O rural O intermediate | |
| Name/affiliation of interviewer(s) | |  | |
| Date, Time (hh-hh), Location | |  | |
|  | **Suggested questions – actual questions should always be adapted to suit the particular case and incorporate any prior knowledge about the interviewee** | | **Remarks** |
|  | 1. ***Brief introduction to the PERISCOPE project and the interviewer*** | |  |
|  | 1. ***Information on goal of the interview*** *Gathering implicit knowledge on aspects that are not already well-covered in the literature – focus on lessons learned to improve preparedness* | |  |
|  | 1. *Signing and exchange of the* ***informed consent form*** *(if not exchanged electronically beforehand)* | |  |
|  | 1. In which ways was your **everyday work** as a [XX] **affected** by the COVID-19 pandemic?  What did you perceive to be the **greatest challenges** in this regard? | | *XX: physician, nurse, etc.*  *In the interest of readability, all questions are formulated in past tense. You may also refer to the present situation if applicable.*  *For all questions: You can refer to experiences from different pandemic phases (e.g., regarding availability of tests, vaccinations, treatment knowledge), but please do not go through them wave by wave.* |
| *Service delivery: supply side* | 1. In which ways were **quality and intensity of care** affected (*acute care/chronic care/administrative consultations**)? Which problems did you observe in this regard **over the course of the pandemic**?  Were **specific patient groups** (specific diseases or sociodemographic groups) particularly affected by problems in service delivery? If yes, which groups and in which ways were they affected?  Which **measures** to tackle these problems did you experience as **most** helpful?   *E.g., measures by government, social insurance, professional association, but also measures taken by the person/institution themselves*  In your view, what **would have been suitable alternative/additional measures** to improve service delivery? - **Who** should have taken these measures? | | *Interviews should preferably address topics that are not already well-covered in the literature. Topics marked in green (=not yet well-covered) should therefore be prioritised over topics marked in red. I.e., if interview person raises one of the topics marked in red, please redirect the conversation to a different topic.*  **Please make sure that all three areas are covered. By “administrative consultations”, we mean services like issuance of prescriptions or sick notes.* |
|  | In your view, which measures should be taken to prepare the primary care sector for similar **future pandemics/crises**?  *Possible topics to be discussed:*   - *Problems in relation to restricted supply (closed practices, reduced opening hours)* - *Fear of getting infected oneself or infecting others* - *Implementation of COVID-specific guidelines* - *Interface management with secondary care and long-term care* - *Resources: e.g., staff (quarantine, care duties), practice facilities (e.g., separate waiting rooms for infectious patients), protective equipment* - *Practice organisation: e.g., separate consultation hours for infectious patients, changes due to staff shortages, remote provision of services (e.g., teleconsultations, e-prescriptions, sick notes)* - *Experience with additional/new services: e.g., telehealth, teleconsultations* | |  |
| *Non-COVID service delivery: demand side* | 1. In which ways was **patient demand** for primary care services affected? Which problems did you observe in this regard **over the course of the pandemic**?  Were **specific patient groups** (specific diseases or sociodemographic groups) particularly affected? If yes, which groups?  Which **measures** to tackle these problems did you experience as **most** helpful?  In your view, what **would have been suitable alternative/additional measures** to overcome these problems? **Who** should have taken these measures?   *Possible topics to be discussed:*   - *Reasons for changes in service use: e.g., fear of getting infected, logistic challenges* - *Acceptance of new ways of service delivery (e.g., telehealth)* - *Continuity of care for chronic patients* | | *Focus should be placed on measures, as problems are already well-covered in literature.* |
| *Service delivery for COVID patients* | 1. Please describe the **typical patient pathway of a COVID patient** who requires primary care services. Which problems did you observe in service delivery for COVID patients **over the course of the pandemic**?  Which **measures** to tackle these problems did you experience as **most** helpful?  In your view, what **would have been suitable alternative/additional measures** to overcome these problems? **Who** should have taken these measures? | |  |
| *Guidelines/ regulations for primary care* | 1. How do you evaluate the various COVID-specific **guidelines and regulations** that were relevant for primary care, both from authorities and from professional associations *(concerning their feasibility, practicability, timeliness,…)*?  How did this **change** over time?  If you had had a say in the development of guidelines/regulations for primary care, what would you have **done in the same way** and what would you have **done differently**? | | *This question is not prioritised – please only ask if sufficient time is left.* |
| *Information* | 1. How do you evaluate the information **you** and **your patients** received *(regarding e.g., guidelines/regulations, infection control)*? What worked well and which problems did you observe in this regard **over the course of the pandemic** *(e.g., poor communication, missing guidelines, conflicting information)*?  Which **measures** directed at information did you experience as **most** helpful?  In your view, what **would have been suitable measures** to improve the flow of information? **Who** should have taken these measures? | | *This question is not prioritised – please only ask if sufficient time is left.* |
| *Learnings/ outlook* | 1. All in all, what do you perceive as the **most important learnings** from the pandemic (1) for **your own future practice** and (2) for the **primary care sector as a whole**? | |  |

Table: Individual characteristics of interview partners in the five country case studies

| ID | Country | Age | Gender | Medical profession | Practice type | Region | Area | Duration of interview  (hh:mm) |
| --- | --- | --- | --- | --- | --- | --- | --- | --- |
| AUT_IP1 | Austria | 61-70 | Male | GP | Group practice | Salzburg | Rural | 00:46 |
| AUT_IP2 | Austria | 41-50 | Male | GP | Group practice | Styria | Urban | 01:00 |
| AUT_IP3 | Austria | 51-60 | Male | GP | Single practice | Styria | Rural | 00:43 |
| AUT_IP4 | Austria | 61-70 | Female | GP | Single practice | Lower Austria | Rural | 01:10 |
| AUT_IP5 | Austria | 41-50 | Male | GP | Group practice | Upper Austria | Urban | 00:56 |
| DNK_IP1 | Denmark | 51-60 | Male | GP | Single practice | Capital Region of Denmark | Urban | 00:49 |
| DNK_IP2 | Denmark | 61-70 | Female | GP | Group practice | Region Zealand | Mixed | 00:53 |
| DNK_IP3 | Denmark | 51-60 | Female | GP | Group practice | Region of Southern Denmark | Rural | 00:50 |
| DNK_IP4 | Denmark | 41-50 | Male | GP | Group practice | Region Zealand | Urban | 01:07 |
| FRA_IP1 | France | 41-50 | Female | GP | Group practice | Paris | Urban | 01:10 |
| FRA_IP2 | France | 51-60 | Female | Nurse | Group practice | Pays de la Loire | Rural | 00:44 |
| FRA_IP3 | France | 41-50 | Male | GP | 24-hour home doctor service | Sud (ex-PACA) | Urban | 01:09 |
| FRA_IP4 | France | 51-60 | Male | GP | Group practice | Brittany | Rural | 01:07 |
| FRA_IP5 | France | 31-40 | Female | Coordinating nurse | Group practice | Grand Est | Rural | 01:11 |
| HUN_IP1 | Hungary | 41-50 | Male | GP, secretary of the biggest GP association | Single practice | Pest County | Rural | 00:44 |
| HUN_IP2 | Hungary | 51-60 | Male | GP | Single practice | Budapest | Urban | 00:42 |
| HUN_IP3 | Hungary | 61-70 | Male | GP, president of GPs’ Medical College | Single practice | Heves county | Rural | 01:06 |
| HUN_IP4 | Hungary | 41-50 | Female | GP | Single practice | Borsod-Abaúj-Zemplén county | Rural | 01:03 |
| HUN_IP5 | Hungary | 61-70 | Male | GP, secretary of Hungarian Medical Chamber | Single practice | Budapest | Urban | 00:55 |
| HUN_IP6 | Hungary | 61-70 | Male | GP | Singe practice | Bács-Kiskun county | Rural | 01:01 |
| ITA_IP1 | Italy | 21-30 | Female | Family nurse | Group practice | Piedmont | Rural | 00:48 |
| ITA_IP2 | Italy | 41-50 | Female | Physician/ COVID manager | Not applicable^1^ | Latium | Mixed | 00:59 |
| ITA_IP3 | Italy | 41-50 | Female | Physician/ communication manager | Not applicable^1^ | Tuscany | Mixed | 01:08 |
| ITA_IP4 | Italy | 61-70 | Female | Family nurse | Group practice | Piedmont | Urban | 01:03 |
| ITA_IP5 | Italy | 31-40 | Female | Physician/ district manager | Not applicable^1^ | Emilia-Romagna | Mixed | 00:50 |
| ITA_IP6 | Italy | 61-70 | Male | GP | Single practice | Tuscany | Rural | 01:13 |
| ITA_IP7 | Italy | 61-70 | Male | GP/coordinator of Primary Complex Care Unit | Group practice | Calabria | Mixed | 00:44 |
| ITA_IP8 | Italy | 61-70 | Male | Physician/Manager of a nursing home^1^ | Not applicable^1^ | Latium | Rural | 01:11 |
| ITA_IP9 | Italy | 61-70 | Male | GP/coordinator of Primary Complex Care Unit | Group practice | Campania | Rural | 00:52 |
| ITA_IP10 | Italy | 61-70 | Male | Physician/ district manager | Not applicable^1^ | Sicily/Piedmont | Mixed | 01:12 |
| ITA_IP11 | Italy | 61-70 | Female | Physician/ district manager | Not applicable^1^ | Marche | Rural | 00:53 |

Note: mixed area = area with rural and urban parts
1 Interviewee is active in a management position
